# The association between empirical dietary inflammatory pattern and colorectal cancer risk: a case-control study

**DOI:** 10.1186/s40795-023-00797-8

**Published:** 2023-11-23

**Authors:** Zainab Shateri, Maede Makhtoomi, Fatemeh Mansouri, Milad Rajabzadeh-dehkordi, Mehran Nouri, Bahram Rashidkhani

**Affiliations:** 1 Department of Nutrition and Biochemistry, School of Medicine, Ilam University of Medical Sciences, Ilam, Iran; 2 Students’ Research Committee, Shiraz University of Medical Sciences, Shiraz, Iran; 3 Department of Community Nutrition, School of Nutrition and Food Sciences, Shiraz University of Medical Sciences, Shiraz, Iran; 4 Department of Clinical Nutrition, School of Nutrition and Food Sciences, Shiraz University of Medical Sciences, Shiraz, Iran; 5 Department of Community Nutrition, School of Nutrition and Food Science, Shiraz University of Medical Sciences, Shiraz, Iran; 6 Department of Community Nutrition, Faculty of Nutrition and Food Technology, National Nutrition and Food Technology Research Institute, Shahid Beheshti University of Medical Sciences, Tehran, Iran

**Keywords:** Empirical dietary inflammatory pattern, Dietary inflammatory potential, Dietary inflammation, Colorectal cancer, Iranian

## Abstract

**Background:**

Colorectal cancer (CRC) is the third most common cancer in Iran. Inflammation plays an essential role in developing CRC. A dietary pattern called the empirical dietary inflammatory pattern (EDIP) has recently been designed based on the inflammatory potential of the diet. Therefore, the present study aimed to investigate the impact of EDIP on the risk of CRC.

**Methods:**

The current case-control study was conducted on 142 controls and 71 CRC cases in three general hospitals and Hospital Cancer Organization in Tehran, Iran. We calculated EDIP by a semi-quantitative food frequency questionnaire. The association between EDIP and CRC were evaluated by logistic regression. The level of significance was p < 0.05.

**Results:**

The results revealed that people who were in the highest tertile of the EDIP had higher odds of CRC (in the adjusted model: odds ratio (OR) = 3.74; 95% confidence interval (CI): 1.38–10.14; P = 0.011).

**Conclusion:**

The present study demonstrated the potential role of dietary-induced inflammation in developing CRC. In the current study, an increase in the intake of red meat, processed meats, and refined grains was observed in the higher EDIP tertiles compared to the lower tertiles. Consequently, to decrease the risk of CRC, it is recommended to reduce the consumption of these foods.

## Introduction

Colorectal cancer (CRC) is slow-growing cancer that begins as a tissue growth or tumor in the inner lining of the colon or rectum [1]. CRC is a serious problem in the world’s public health and increasingly affects the population of Asian countries [2]. This is the third most common cancer in Iran [3] and the fourth and third most common in Iranian women and men, respectively [4]. In 2020, 11,942 new cases of CRC were reported in Iran [5].

Inflammation plays an essential role in developing CRC [6]. Specific nutrients and dietary components have been found to influence inflammation [7]. Studies have shown the role of sugar [8] and foods with a high concentration of saturated fat [9] in causing inflammation. On the contrary, it has been shown that tryptophan metabolites [10, 11] and fiber intake [12] can play a role in reducing inflammation. A study in the Iranian population also demonstrated that traditional dietary pattern that are associated with increased consumption of casserole, potato, legumes, hydrogenated fats, whole grains, tea, and refined grains can positively increase interleukin-6 (IL-6) [13]. There are two important indices to describe the inflammatory potential of a diet, including a dietary inflammatory index (DII) and an experimental dietary inflammatory pattern (EDIP) [14, 15]. The DII is essentially a priori index that emphasizes the inflammatory potential of dietary nutrients [16], while EDIP is a posterior index that emphasizes the inflammatory potential of food groups, and this index seems to provide a new dimension of the inflammatory potential of the entire diet of people [17, 18]. In fact, EDIP has been designed based on the inflammatory potential of the diet (according to circulating concentrations of inflammatory biomarkers) [19].

The relationship of EDIP with metabolic syndrome [20], cognitive function [21], and metabolic phenotypes in overweight and obesity [15] has been investigated. However, there are limited studies on the relationship between EDIP and CRC risk [22, 23]. A cohort study aimed at investigating the association between EDIP and the risk of CRC showed a significant positive correlation [22]. Another study showed that the higher the diet’s inflammatory potential, the higher the risk of CRC [24].

Epidemiological studies have not consistently shown an association between the levels of inflammatory markers and the risk of CRC [25]. To the best of our knowledge, there is no study on the effect of EDIP on the risk of CRC among Iranian population. So, the present study aimed to investigate the impact of EDIP on the risk of CRC.

## Methods

The present case-control study was conducted on three hospitals that included Shariati, Imam Hossein, and Ayatollah Taleghani and 19 CRC surgery departments of Imam Khomeini Hospital Cancer Organization in Tehran. The study sample size was calculated based on the odds ratio (OR) = 0.45, α = 0.05, and β = 0.20 based on Terry and et al. study [26].

The newly diagnosis cases with CRC were those who were 40 to 75 years old at the time of study, their diseases were confirmed pathologically. Characteristics of the control group included random selection from the same hospitals and hospitalization for acute and non-neoplastic conditions at the same time, without diet-related chronic diseases. The most common reasons for hospitalization included fractures and sprains, bone and joint disorders, and disc disorders. Each patient with CRC was matched in age and gender with two patients in the control group.

At first, 267 patients (89 cases and 178 controls) were assessed, and 24 participants were excluded by inclusion and exclusion criteria (8 cases and 16 controls). In addition, 10 cases and 20 controls were excluded for incomplete food frequency questionnaire (FFQ), unwillingness, and total energy intake (out of mean ± 3 standard deviations (SDs)). Finally, 71 participants in the case and 142 participants in the control group were included for statistical analyses. This study was approved by the Medical Research and Ethics Committee of Shiraz University of Medical Science (IR.SUMS.SCHEANUT.REC.1401.011). Details of the current study have been previously published [8, 27].

## Dietary assessment

To evaluate dietary intake, we used a semi-quantitative FFQ that included 168 foods and drinks (with standard portion sizes). Based on previous studies, it has been determined that this FFQ has good reliability and reproducibility [28, 29].

The participants stated the frequency of consumption of one meal of each food item daily, weekly, monthly and yearly before CRC diagnosis. Then, the data obtained from their answers were converted into daily consumption frequency. After the portion size of each food item consumed was converted to grams, the consumption amount of each food item in grams was obtained by multiplying the portion size by the number of daily consumption. The edible portion of foods was determined using household measurement guidelines [30]. The nutrient composition of Iranian food data [31] and the United States Department of Agriculture (USDA) food composition data were applied to determine the energy value of food [32]. Total energy intake was estimated by adding the energy value of each food to the FFQ. The Nutritionist IV was applied to calculate energy and intake of nutrient [33].

Tabung et al.‘s study was used to calculate the EDIP score [34]. This study included 18 food items. Diagnostic biomarkers interleukin-6 (IL-6), high-sensitivity C-reactive protein (hs-CRP), and tumor necrosis factor-alpha (TNF-α) were used to make this dietary index. Each item was assigned a specific weight based on its relationship with biomarker levels. We did not include alcoholic beverages such as wine and beer in the calculation of scores because their consumption is not common and may have been underreported due to religious considerations in our study population. Because we did not have any food item as a low-energy drink in our FFQ, this item was also removed from the questionnaire. So, we calculated the EDIP score based on 15 instead of 18, including red meat (lamb or beef), processed meat (sausage), organ meat (calf, beef, or chicken liver), fish (fish or canned tuna), refined grains (white rice, biscuits, white bread, vermicelli or pasta), vegetables (cooked mushrooms, green peppers, mixed vegetables, zucchini, eggplant, cucumber), dark yellow vegetables (squash or carrots), green leafy vegetables (spinach, lettuce or cabbage), tomatoes as a pro-inflammatory group, tea, coffee, high-energy and low-energy drinks (carbonated drinks with sugar, cola with sugar, fruit drinks, fruit juice (apple juice, cantaloupe juice, orange juice or other fruit juices)), snacks (potato chips or crackers), and the anti-inflammatory group of pizza. The proposed weights and inflammatory score of each food groups were multiplied by the average daily intake of each food item, and then all the weight values were added up. Finally, to avoid the large scores, they were all summed up and divided by 1000. A higher score indicates a pro-inflammatory diet in EDIP and vice versa [35].

## Covariates

The case and control groups were interviewed by skilled interviewers. Information obtained from this interview included socio-demographic characteristics, physical activity, family history of CRC, medication information (taking non-steroidal anti-inflammatory drugs (NSAIDs), vitamin/mineral supplements), smoking habits, cooking methods, and dietary intake.

To accurately measure the participants’ weight, they were asked to wear minimal clothing and without shoes, and they were weighed with an accuracy of 0.1 kg. SECA scale (Germany) body measuring device with 0.1 cm accuracy was used for height measurement. Their height was measured while standing without shoes. Recumbent length was acquired for hospitalized patients.

The body mass index (BMI) was calculated as weight (in kg) divided by the square of height (in meters) and classified according to the World Health Organization standard for adults [36]. A valid self-report questionnaire was utilized to measure the level of physical activity of the individuals. This questionnaire contains the level of physical activity of the participant’s metabolic equivalent of task (MET)-hours/day [37]. Physical activity was done based on the activities performed in the year before the CRC diagnosis of the cases or the year before the interview of the controls.

## Statistical analysis

The Kolmogorov-Smirnov test was utilized to evaluate the normality of variables. The chi-square test was used to assess the relationship between classified variables. Mann-Whitney or independent samples T-test was used to determine the association between continuous variables. Also, the Kruskal-Wallis test was used to evaluate food group intake across the tertiles of EDIP. The association between EDIP and CRC were evaluated by logistic regression and adjusted for energy and fiber intake, smoking, physical activity, BMI, family history of CRC, household income, education level, the common way of cooking meat, and taking ibuprofen, aspirin, acetaminophen and mineral supplement use. SPSS software (version 26.0) was used for statistical analysis. The level of significance was p < 0.05.

## Results

The baseline characteristics of study population are shown in Table 1. As can be seen, the mean of EDPI score, BMI, and age in the case group was higher than in the control group but it was not statistically significant. Also, the energy intake and physical activity were not significantly different between the case and control groups. However, fiber intake (P < 0.001), family history of CRC (P = 0.0017) and taking aspirin (P = 0.016), acetaminophen (P = 0.004) and mineral supplement (P = 0.015) illustrated a significant differences between the case and control groups.

**Table 1:** Baseline characteristics of study population

Variables	Cases (n = 71)	Controls (n = 142)	P-value
**Quantitative Variables**			
Age (year) ^1^	58.2 ± 10.4	57.7 ± 10.4	0.746
Physical activity (MET-h/day) ^1^	36.8 ± 3.6	36.7 ± 4.8	0.932
BMI (kg/m^2^) ^1^	27.6 ± 4.2	26.6 ± 4.2	0.362
EDIP score ^1^	2.1 ± 0.8	1.9 ± 0.8	0.076
Energy (kcal/day) ^1^	2262.3 ± 450.1	2255.2 ± 341.2	0.908
Fiber (g/day) ^1^	18.9 ± 2.3	20.4 ± 3.1	**< 0.001**
Income (dollar) ^2^	393.0 (253.0)	402.0 (302.0)	0.206
**Qualitative Variables**			
Smoking ^3^ Never Former Current	57 (80.2) 8 (11.3) 6 (8.5)	101 (70.1) 15 (10.6) 26 (18.3)	0.164
Education ^3^ No formal education Elementary Junior/Senior high school Diploma/College/University	28 (39.3) 22 (31.0) 7 (9.9) 14 (19.7)	36 (25.4) 45 (31.6) 19 (13.4) 42 (29.6)	0.147
Family history of CRC, yes ^3^	7 (9.9)	3 (2.1)	**0.017**
Aspirin, yes ^3^	1 (1.4)	14 (9.9)	**0.016**
Acetaminophen ^3^	4 (5.6)	28 (19.7)	**0.004**
Ibuprofen, yes ^2^	5 (7.0)	22 (15.5)	0.059
Mineral supplement use, yes ^3^	8 (11.3)	35 (24.6)	**0.015**
Common ways of cooking meat ^3^ Raw / Fresh Boiled Fried, Fried / Frozen	29 (40.8) 8 (11.3) 34 (47.9)	78 (54.9) 18 (12.7) 46 (32.4)	0.083

Using chi-square test for categorical and Mann-Whitney or independent samples T-test for continuous variables

MET: metabolic equivalent of task, BMI: body mass index, EDIP: empirical dietary inflammatory pattern

^1^Values are mean ± SD

^2^Values are median (IQR)

^3^Values are number (percent)

Based on Table 2, among the variables, tomatoes (P = 0.021), red meat (P = 0.011), processed meat (P = 0.021), refined grains (P < 0.001), and pizza (P < 0.001) had significant differences across the tertiles of EDIP.

**Table 2:** Food group intake across the tertiles of EDIP

Variables	T1 (n = 71)	T2 (n = 71)	T3 (n = 71)	P-value
	Median (IQR)	Median (IQR)	Median (IQR)	
**Pro-inflammatory group**				
Dark or yellow vegetable (serving/day)	0.15 (0.23)	0.15 (0.23)	0.15 (0.23)	0.227
Other vegetable (serving/day)	0.73 (0.74)	1.01 (0.82)	1.01 (0.83)	0.058
Tomatoes (serving/day)	0.79 (0.47)	0.80 (0.47)	0.80 (0.85)	**0.021**
Refined grains (serving/day)	4.77 (3.20)	7.67 (3.16)	11.76 (4.92)	**< 0.001**
Red meat (serving/day)	0.30 (0.24)	0.38 (0.34)	0.38 (0.33)	**0.011**
Processed meat (serving/day)	0.05 (0.17)	0.05 (0.13)	0.09 (0.23)	**0.021**
Organ meat (serving/day)	0.004 (0.01)	0.004 (0.02)	0.004 (0.02)	0.599
Fish (serving/day)	0.15 (0.68)	0.16 (0.18)	0.16 (0.17)	0.686
**Anti-inflammatory group**				
High-energy beverage (serving/day)	0.05 (0.12)	0.05 (0.14)	0.15 (0.27)	0.075
Tea (serving/day)	4.08 (3.06)	3.06 (2.55)	3.06 (2.04)	0.424
Coffee (serving/day)	0.00 (0.04)	0.00 (0.01)	0.00 (0.01)	0.527
Snacks (serving/day)	0.67 (0.97)	0.55 (0.78)	0.57 (0.55)	0.150
Fruit juice (serving/day)	0.00 (0.11)	0.08 (0.16)	0.04 (0.18)	0.533
Pizza (serving/day)	0.20 (0.28)	0.09 (0.15)	0.10 (0.11)	**< 0.001**

Using Kruskal-Wallis test

Values are median (IQR)

EDIP: empirical dietary inflammatory pattern, IQR: interquartile range

Table 3 displays the association between EDPI and CRC. The second logistic regression model was adjusted for energy and fiber intake, smoking, physical activity, BMI, family history of CRC, the common way of cooking meat, and taking ibuprofen, aspirin, acetaminophen and mineral supplement use. According to this model, the chance of developing CRC in the last tertile of EDIP was significantly higher than in the first tertile (OR = 3.74; 95% confidence interval (CI): 1.38–10.14; P = 0.011).

**Table 3:** Association between EDIP and colorectal cancer

Tertiles of EDIP	Case/Control	Model 1		Model 2	
		OR	95% CI	OR	95% CI
T_1_ (≤ 0.37)	21/50	1.00	Ref.	1.00	Ref.
T_2_ (0.38–0.69)	19/52	0.87	0.41–1.80	0.98	0.38–2.50
T_3_ (≥ 0.70)	31/40	1.84	0.92–3.68	**3.74**	**1.38–10.14**
P_trend_		0.076		**0.011**	

Obtained from logistic regression

These values are odds ratio (95% CIs)

Significant values are shown in bold

EDIP: empirical dietary inflammatory pattern

Model 1: crude model

Model 2: adjusted for energy and fiber intake, smoking, physical activity, BMI, family history of CRC, household income, education level, the common way of cooking meat, and taking ibuprofen, aspirin, acetaminophen and mineral supplement

## Discussion

In the current case-control study, we examined the association between the inflammatory potential of the diet, as measured by EDIP, and developing CRC odds. The findings showed that greater adherence to EDIP was associated with a higher odds of CRC. Also, the results indicated that more adherence to this dietary pattern was associated with more intake of red meat, processed meats, and refined grains. Therefore, limiting the consumption of the mentioned foods may play a role in reducing the odds of CRC.

EDIP was recently developed, and its validity was evaluated in two independent cohorts of men and women [34]. It has been shown that EDIP could remarkably anticipate the levels of inflammatory markers [34]. This inflammation-related dietary pattern reveals the inflammatory impacts of foods in a total diet. Also, the development of an inflammatory index based on food groups make possible the provision of nutritional recommendations to alleviate diet-induced inflammation compared to using a single nutrient or food [15].

As previously mentioned, the findings showed a positive association between EDIP and CRC odds. Our findings are in line with previous research. Tabung et al. demonstrated that the higher EDIP was positively related to the CRC risk [22]. Also, another study by Tabung et al. revealed that higher dietary inflammatory index (DII) scores were associated with an increased risk of CRC [38]. Moreover, a case-control study by Shivappa et al. indicated a positive relationship between the inflammatory potential of the diet and the risk of CRC [39]. A meta-analysis study on case-control studies also demonstrated that greater adherence to DII was associated with an increased risk of CRC (relative risk = 1.27; 95% CI: 1.16–1.38) [40].

The precise mechanisms by which inflammation leads to CRC are gradually becoming elucidated [41]. The transcription factor nuclear factor kappa B (NF-κB), which is involved in many inflammatory pathways, is one of the well-known mechanisms by which chronic inflammation causes cancer [42]. TNF-α also plays a role in developing CRC. TNF-α, a pro-inflammatory cytokine, has a positive role in activating NF-κB [43]. Also, the role of pro-inflammatory mediators such as cyclooxygenase-2 (COX-2) and prostaglandin E2 (PGE2) has been shown in cancer progression. Moreover, COX-2-derived PGE2 increases the growth of colonic tumor by silencing DNA repair genes and specific tumor suppressors by DNA methylation in colonic epithelial tumor cells [44].

As shown in the current study, higher EDIP scores were related to more intake of red meat and processed meats. Processed meats and various components of red have been proposed to contribute to chronic inflammation and the risk of disease, such as nitrosamines, heme iron, and advanced glycation end products [45]. Also, fat intake is high in a diet rich in processed meats and red meat. Studies have indicated the role of dietary fat in developing CRC [46, 47]. High-fat intake stimulates the secretion of secondary bile acids in the intestine. The role of bile acids in tumor formation has been shown [48]. Moreover, heterocyclic amines (HCAs) and polycyclic aromatic hydrocarbons (PAHs) are other compounds that induce carcinogenesis through mutation induction. These compounds are found in red meat and processed meats [49–51].

Also, more intake of refined grains was observed in people who had more adherence to EDIP. An association between increased consumption of refined grains and the risk of CRC has been shown [52]. Chatenoud et al. reported that the highest intake of refined grains compared to the lowest intake was associated with ORs of 1.3 for rectal cancer and 1.5 for colon cancer [53]. Further, a study by Levi et al. indicated a positive association between refined grains and CRC (OR = 1.32 for increasing one serving/day) [54].

The role of other dietary patterns on inflammation has also been investigated. A systematic review study on interventional and observational studies showed that there is an inverse relationship between dietary patterns such as Dietary Approaches to Stop Hypertension (DASH) diets, and the Mediterranean diets and pro-inflammatory biomarkers [55]. Also, in a prospective cohort study on the older adults, it was shown that a dietary pattern high in vegetables, fruits, fish, poultry, whole grains, and low-fat dairy products is associated with a reduction in systemic inflammation [56]. Moreover, Nettleton et al. revealed an inverse relationship between a ‘vegetables and fish’ pattern and IL-6, an inverse relationship between a ‘whole grains and fruit’ pattern and CRP and IL-6, and a positive association between a ‘fats and processed meats’ pattern and CRP and IL-6 [57]. In general, based on studies, it can be found that a “healthy” dietary pattern, high in fish, poultry, vegetables, whole grains, and fruits and low in sweetened beverages, sweets, desserts, red and processed meat, high-fat dairy products, and refined grains are associated with lower levels of systemic inflammation compared to other dietary patterns [56].

The strengths of the present study are that we controlled for several potential confounding factors for examining the relationship between EDIP and CRC risk. A valid and reliable questionnaire was also used to collect data. However, although the effect of several confounding variables was adjusted, there might be other confounding variables that we did not consider in the study. Also, like other case-control studies that examine dietary intake before diagnosis, this study may have recall bias.

## Conclusion

This case-control study demonstrated the potential role of dietary-induced inflammation in developing CRC. In the present study, an increase in the intake of red meat, processed meats, and refined grains was observed in the higher EDIP tertiles compared to the lower tertiles. Consequently, to decrease the risk of CRC, it is recommended to reduce their consumption. Also, it is possible participants change their diet after diagnosis of each diseases and improve their usual diet, for this reason, dietary intake assessed after CRC diagnosis and newly CRC diagnostic cases were selected. Since this study was conducted in Iran, the results cannot be generalized to other populations. Therefore, more research is necessary to clarify the role of this dietary pattern in CRC risk.

## Data Availability

To protect study participant privacy, data cannot be shared openly, but data are available through a reasonable request from the corresponding author.
